# The male genital system of the cellar spider *Pholcus phalangioides *(Fuesslin, 1775) (Pholcidae, Araneae): development of spermatozoa and seminal secretion

**DOI:** 10.1186/1742-9994-2-12

**Published:** 2005-06-29

**Authors:** Peter Michalik, Gabriele Uhl

**Affiliations:** 1 Zoologisches Institut und Museum, Ernst-Moritz-Arndt-Universität, J.-S.-Bach-Straße 11/12, D-17489 Greifswald, Germany; 2 Institut für Zoologie, Universität Bonn, Endenicher Allee 11-13, D-53115 Bonn, Germany

## Abstract

**Background:**

Most arthropods pass through several molting stages (instars) before reaching sexual maturity. In spiders, very little is known about the male genital system, its development and seminal secretions. For example, it is unknown whether spermatozoa exist prior to-, or only after the final molt. Likewise, it is unclear whether sperm are produced throughout male adulthood or only once in a lifetime, as is whether seminal secretions contain factors capable of manipulating female behavior. In order to shed light on these aspects of the reproductive biology of spiders, we investigated the male genital system of the common cellar spider *Pholcus phalangioides*, with special emphasis on its development and seminal secretions.

**Results:**

Testes already display all stages of spermatogenesis in subadult males (about four weeks before the final molt). Their vasa deferentia possess proximally a very voluminous lumen containing dense seminal fluid and few spermatozoa, whereas the distal part is seemingly devoid of contents. Spermatoza of *P. phalangioides *are typical cleistospermia with individual secretion sheaths. In male stages approximately two weeks prior to the final molt, the lumina of the testes are wider and filled with a dense secretion. The wide, proximal portion of the vasa deferentia is filled with secretion and a large number of spermatozoa, and the narrow distal part also contains secretion. In adult males, the wide lumina of the testes are packed with spermatozoa and secretions. The latter are produced by the somatic cells that bear microvilli and contain many vesicles. The lumina of the vasa deferentia are narrow and filled with spermatozoa and secretions. We could identify a dense matrix of secretion consisting of mucosubstances and at least three types of secretion droplets, likely consisting of proteinaceous substances.

**Conclusion:**

This study reveals that spermatogenesis begins weeks before maturity and takes place continuously in the long-lived males of *P. phalangioides*. Possible functions of the various types of secretion in the seminal fluid and previously investigated female secretions are discussed in the light of sexual selection.

## Background

When and for how long males produce sperm very likely depends on the mating system of the species in question. Long-lived species in which males can expect several matings probably continue to produce sperm throughout their lifetime. On the other hand, in species with a short reproductive time window or in species that show a considerable drop in female receptivity after a single mating, a male is expected to allocate all resources into sperm production for a single mating. For spiders, both evolutionary scenarios are conceivable: in sexually cannibalistic spiders, males load each of their copulatory organs, the pedipalps, only once and cease to produce sperm after the final molt, which seems to be a consequence of the high incidence of monogamy that is forced upon the male by female cannibalistic attacks [[1]; PM personal observation], whereas in most spider species, males can expect more than one mating. In our focal species, *Pholcus phalangioides*, adult males are especially long-lived, with life-spans of up to a year [GU personal observation]. In this group of spiders sperm should be produced throughout a male's lifetime.

During mating, males not only transfer sperm, but also seminal fluid [2-6]. The seminal fluid of *Drosophila melanogaster *contains over 80 proteins and peptides. The few substances that have been identified have marked effects on the reproductive success of males and females: seminal fluid proteins and peptides can decrease female receptivity, increase egg production, facilitate sperm storage, and are necessary for sperm transfer and success in sperm competition. Moreover, antimicrobial agents in the secretions ensure that the female reproductive tract is a hospitable environment during sperm transfer and storage. In some species, even noxious chemicals are transferred and incorporated into developing eggs to protect them from predators and pathogens [reviews: [4-7]]. Similar functions have been reported from other insects such as butterflies [8] and crickets [9]. In most insects, males produce secretions in separate accessory glands that can be dissected to characterize their products. However, male spiders have not been reported to possess separate accessory glands that are directly connected to the genital tract. The production of seminal secretion is thus very likely to occur within the testes. However, it is conceivable that the effects of the seminal secretions on the reproductive success of males and females may be similar in spiders and insects despite their different origin.

The shape of the male genital system in spiders differs enormously across spider taxa, but usually consists of two thick strands of testes, continuing in the thin convoluted vasa deferentia, which fuse distally to form the unpaired ductus ejaculatorius that opens into the genital opening located in the epigastric furrow [for entelegyne araneomorph spiders: [10-13]; but see [14] for Theraphosidae]. Generally, the male genital system in spiders is embedded in the midgut gland and the testes often extend deeply into the opisthosoma. Additional multicellular glands present in male spiders are the so-called epiandrous (=epigastric) glands, which seem to provide part of the sperm web and release their acinous substances near the genital opening through special spigots (=fusules) [15-17]. For some spider families unicellular glands that open into the epigastric furrow via individual ducts are described, but it is unknown whether the glandular secretion is added to the sperm mass [17,18]. Unpublished work supports the notion that there is a great diversity of secretions in the male genital tract of spiders, the origin and functions of which are unknown [[10]; PM, GU personal observation].

In the present study, we briefly describe the genital system of the haplogyne spider *P. phalangioides*. We investigate the organisation and development of the male genital system, the ultrastructure of the different parts, and give a first characterization of the secretions produced in the genital system. Finally, we present some additional findings on spermatogenesis and spermatozoa that complement previous investigations [19-21].

## Results

**Figure 1:**
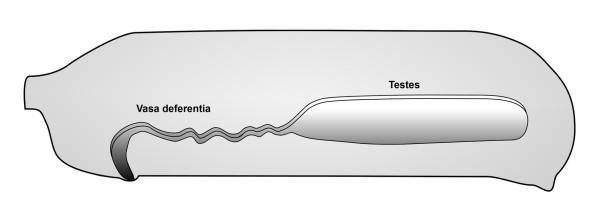
Schematic drawing of the male genital system of *Pholcus phalangioides*.

**Figure 2:**
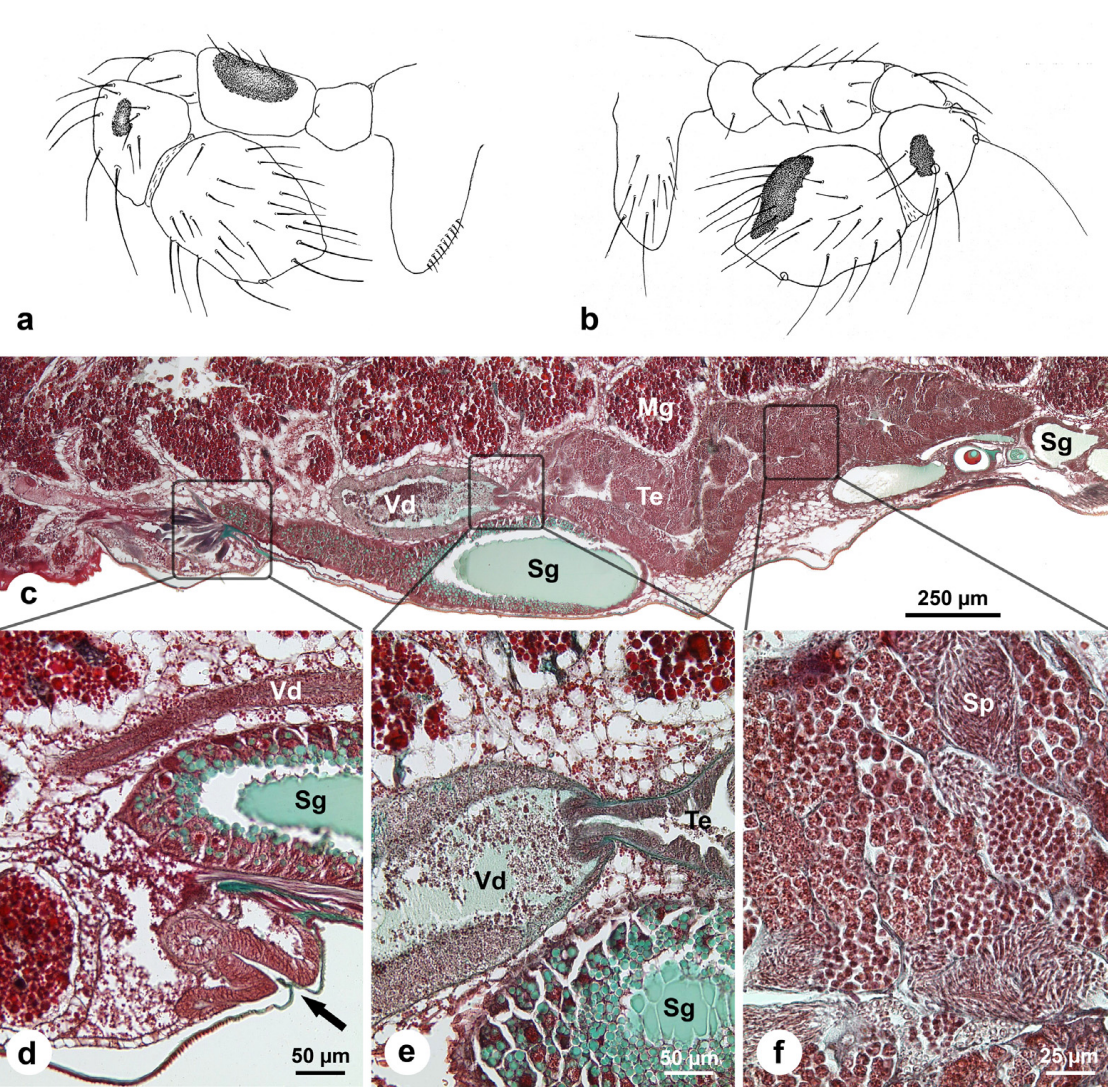
**Male genital system of *Pholcus phalangioides *in subadult stage 1 (about four weeks before final molt)**. (a-b): Drawings of the palpal organ from medial (a) and lateral (b) view to characterize the age of the observed males. (c): Longitudinal section of the male genital system (anterior = left). The vas deferens and large part of the testis are bordered ventrally by silk glands. The proximal part of the lumen of the vas deferens is very voluminous and filled with some spermatozoa and dense secretion. (d): Longitudinal section in the region of the genital opening (arrow). In this part, the vas deferens has a very narrow lumen without recognizable content. (e): Transition between testis and vas deferens. The connection between these two parts has a valve-like appearance. The vas deferens possesses a thick epithelium and is filled with dense secretion and some spermatozoa. (f): Section of the testis. Note germ cells at different stages of spermatogenesis in cysts. The branched lumen is very narrow and a secretion is not yet recognizable. *Mg*, midgut gland; *Sg*, silk gland; *Sp*, spermatids; *Te*, testis; *Vd*, vas deferens.

**Figure 3:**
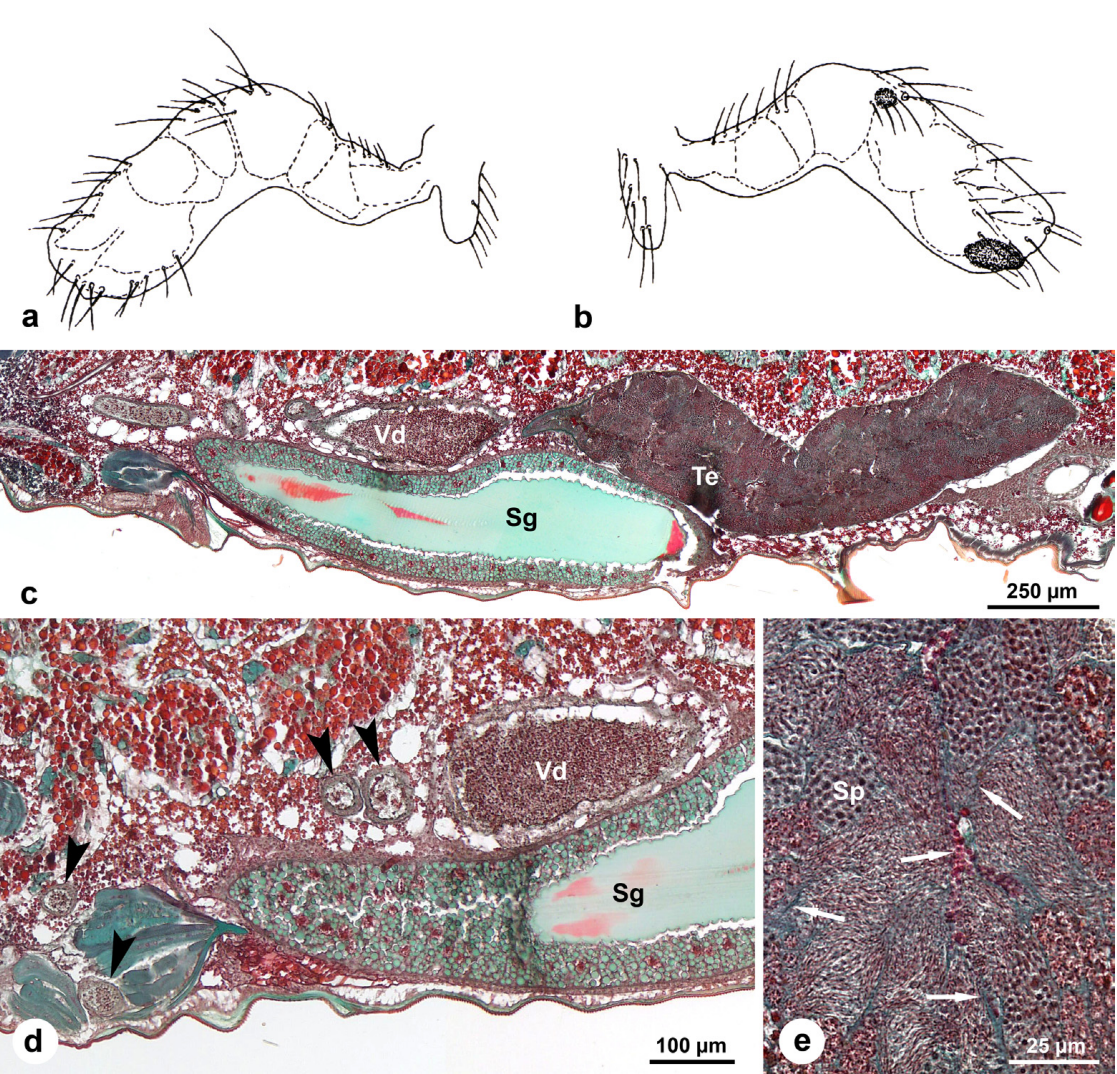
**Male genital system of *Pholcus phalangioides *in subadult stage 2 (about two weeks before final molt)**. (a-b): Drawings of the palpal organ from medial (a) and lateral (b) view to characterize the age of the observed males. (c): Longitudinal section of the male genital system. Note the different staining of cells compared with Fig. 2c resulting from a higher secretory activity (see Fig. 3e). (d): Longitudinal section of the vas deferens. The voluminous proximal part is completely filled with spermatozoa and secretion. Towards the distal part, the lumen becomes narrow but is also filled with seminal fluid (arrows). (e): Section of the testis. Between the cysts the narrow lumen contains homogenous secretion characterized by the blue-green color. *Sg*, silk gland; *Sp*, spermatids; *Te*, testis; *Vd*, vas deferens.

**Figure 4:**
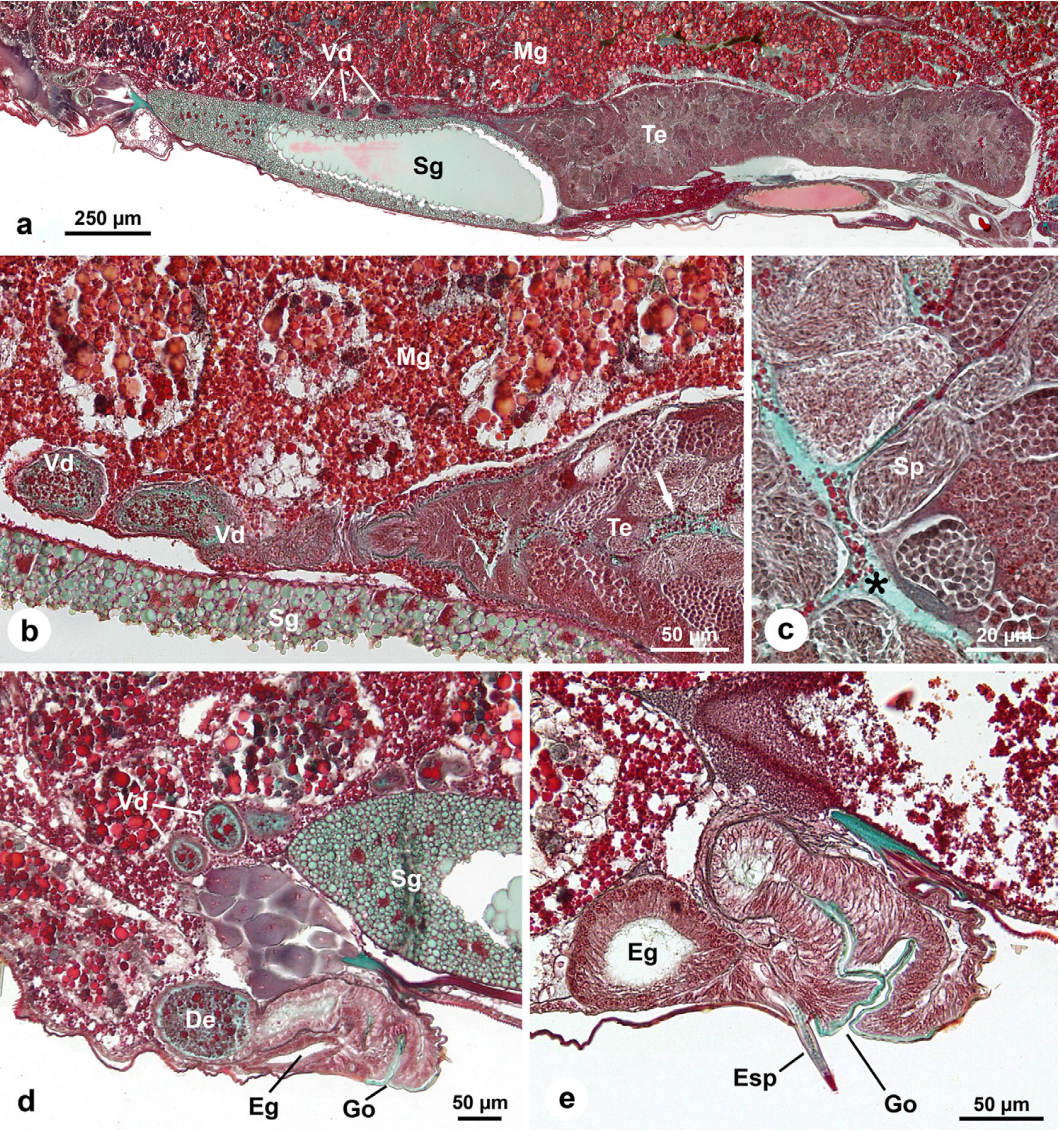
**Male genital system of adult *Pholcus phalangioides *(one day after final molt)**. (a): Longitudinal section of the male genital system. The convoluted vas deferens possesses a thin tube-like shape. Within the testis the different stages of spermatogenesis, spermatogonia (dark) and later stages of spermatogenesis near the center (bright) are clearly visible. (b): Transition between testis and vas deferens. The thin vas deferens is filled with spermatozoa and different kinds of secretion (c.f. Fig. 6). (c): Section of the testis. The lumen is very wide and contains different secretions. Note the secretion droplets (asterisk) and the different cysts of spermatids. (d): Near the genital opening the vasa deferentia fuse to form the ductus ejaculatorius which accumulates large amounts of seminal fluid. (e): Longitudinal section through the genital opening, bordered by the cuticle. The epiandrous apparatus is located in front of the genital opening. Note the epiandrous gland and its secretion within the spigot. *De*, ductus ejaculatorius; *Eg*, epiandrous gland; *Esp*, epiandrous spigot; *Go*, genital opening; *Mg*, midgut gland; *Sg*, silk gland; *Sp*, spermatids; *Te*, testis; *Vd*, vas deferens.

The male genital system of *P. phalangioides *consists of one pair of massive testes and convoluted vasa deferentia which become thicker near the genital opening and fuse distally to form the ductus ejaculatorius (Fig. 1). The genital tract is located ventrally in the opisthosoma and the testes extend as far as to the spinning apparatus (Figs. 2c, 3c, 4a). Parts of the testes and the vasa deferentia are bordered ventrally by the ampullate silk glands (Figs. 2c, 3c, 4a). The genital system is surrounded by extensions of the midgut gland (Figs. 2c, 3c, 4a).

### Developmental Stages of the Male Genital System

#### Stage 1 (about four weeks before the final molt)

These young males are characterized by palpal organs that are bent in the joint of the tibia and patella (Figs. 2a,b). Dark sclerotized areas are present on the femur and tibia. The tarsus has a broad shape without any signs of internal structures or appendages as present in adult males (Figs. 2a,b).

The longitudinal section through the opisthosoma (Fig. 2c) shows that the dimensions of the testis are similar to the final dimensions in adult males (compare Fig. 4a), and all stages of spermatogenesis are observable (Fig. 2f). Spermatogenesis occurs in cysts containing spermatids of the same developmental stage (Fig. 2f, see also Figs. 3e, 4c). The secretory activity within the testis is very low as indicated by the absence of the dark green secretion visible in subadult stage 2 and adults (see below).

The most distal part of the testis becomes thinner and opens into the vas deferens, forming a valve-like structure (Fig. 2e). The proximal part of the vas deferens possesses a thick epithelium and has an extensive lumen that is filled with spermatozoa embedded in a bright green secretion (Figs. 2c,e). In the more distal parts the lumen, the vas deferens is very narrow. It contains neither spermatozoa nor any other recognizable substance (Fig. 2d).

#### Stage 2 (about two weeks before the final molt)

In this stage the palpal organs are only slightly bent. The tarsus is extended and the internal structures known from the palps of adult males are visible through the cuticle (Figs. 3a,b). Dark sclerotized areas are present on the distal and lateral side of the tarsus (Figs. 3a,b).

The section in Figure 3c displays a similar organization as that in subadult males of stage 1. The testis contains all stages of spermatogenesis (Fig. 3e). Lumina with dark green secretion and several spots with red, roundish secretion are present between the cysts of spermatid (Fig. 3e, arrows). This secretion is produced by the somatic cells of the testis (see below).

The extensive proximal part of the vas deferens is almost completely filled with spermatozoa and secretions (Figs. 3c,d). Distally, the vas deferens becomes thinner and is shaped as a convoluted tube (Figs. 3c,d). Within the lumen a similar material as in the proximal part is present (Fig. 3d, arrows).

#### Adult Stage

**Figure 5:**
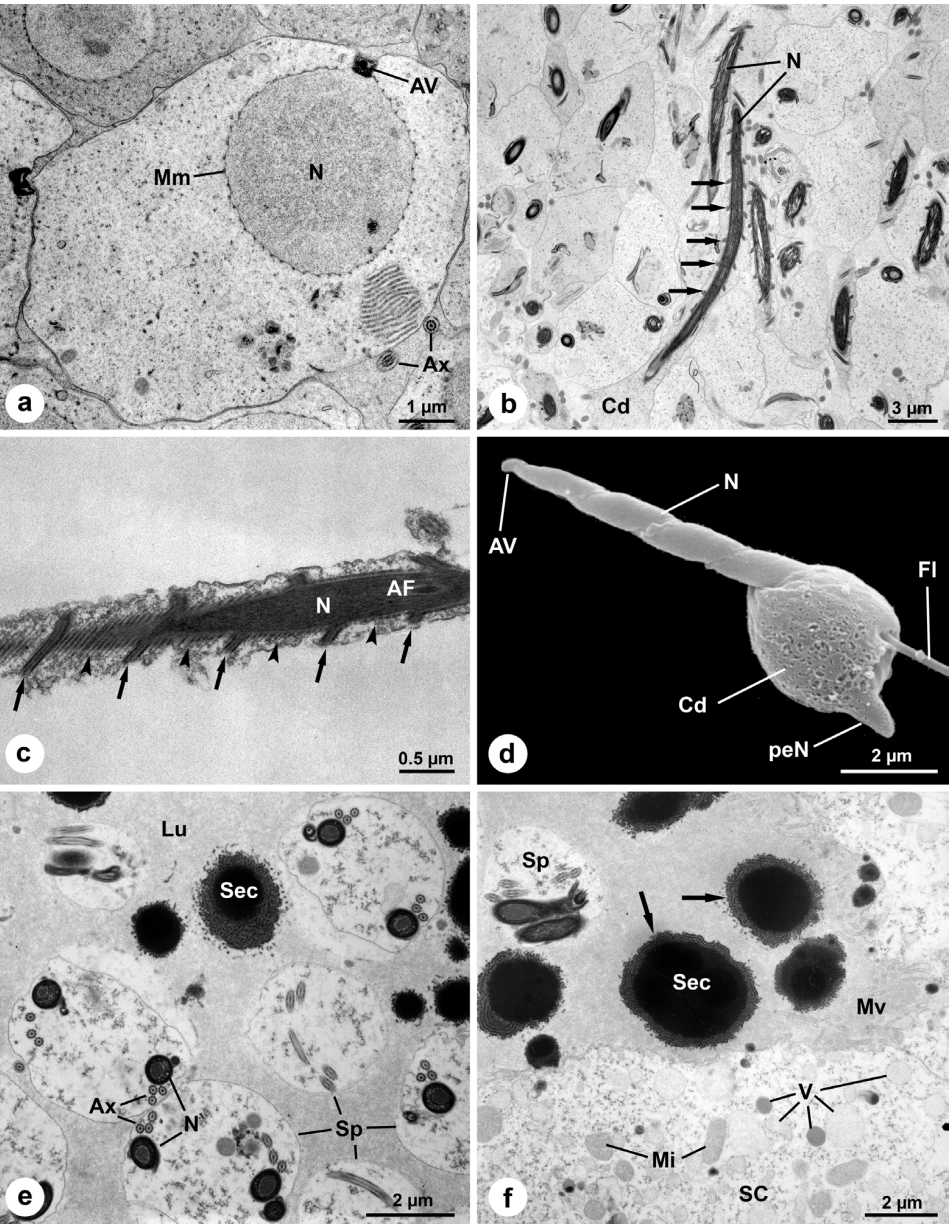
**Ultrastructure of spermatozoa and testis of *Pholcus phalangioides***. (a): Early spermatid. The nucleus is surrounded by a manchette of microtubules with a peculiar regular pattern. The nuclear envelope possesses a wave-like outline. The cytoplasm in front of the acrosomal vacuole at the anterior pole of the nucleus is indented. Note the annulate lamellae near the nucleus. (b): Late spermatids. The chromatin of the nucleus is almost completely condensed. The nucleus possesses a very elongated shape with a conspicuous helical band whirling around it (arrows). (c): Detail of the nucleus. The helical band of the nucleus is very thin and bordered by microtubules (arrows). Between the band, the dense manchette of microtubules is conspicuous (arrowheads). (d): Scanning electron micrograph of a late spermatid. Note the extensive cytoplasm drop at the posterior end of the spermatid. The helical character of the nucleus is clearly visible. (e): Lumen of the testis with coiled spermatids. The coiling occurs at the end of spermiogenesis within the testis. Between the coiled spermatids several secretion droplets are visible. (f): The somatic cells possess many different vesicles and are bordered apically by microvilli. They continuously produce the large secretion droplets (arrows). *AF*, acrosomal filament; *AV*, acrosomal vacuole; *Ax*, axoneme; *Cd*, cytoplasm droplet; *Fl*, flagellum; *Lu*, lumen; *Mi*, mitochondria; *Mv*, microvilli; *N*, nucleus; *peN*, postcentriolar elongation of the nucleus; *SC*, somatic cell; *Sec*, secretion droplet; *Sp*, spermatids.

The fully developed male genital system is also characterized by tube-like testes and thin convoluted vasa deferentia (Figs. 1, 4a). Within the testes all stages of spermatogenesis are visible (Figs. 4a,b). The spermatozoa are coiled, which is typical of spiders (Fig. 5e). In comparison to the subadult stages, the lumen of the adult testis seems wider and is filled with a bright green secretion matrix and red, roundish secretion droplets of different sizes (Fig. 4c).

**Figure 6:**
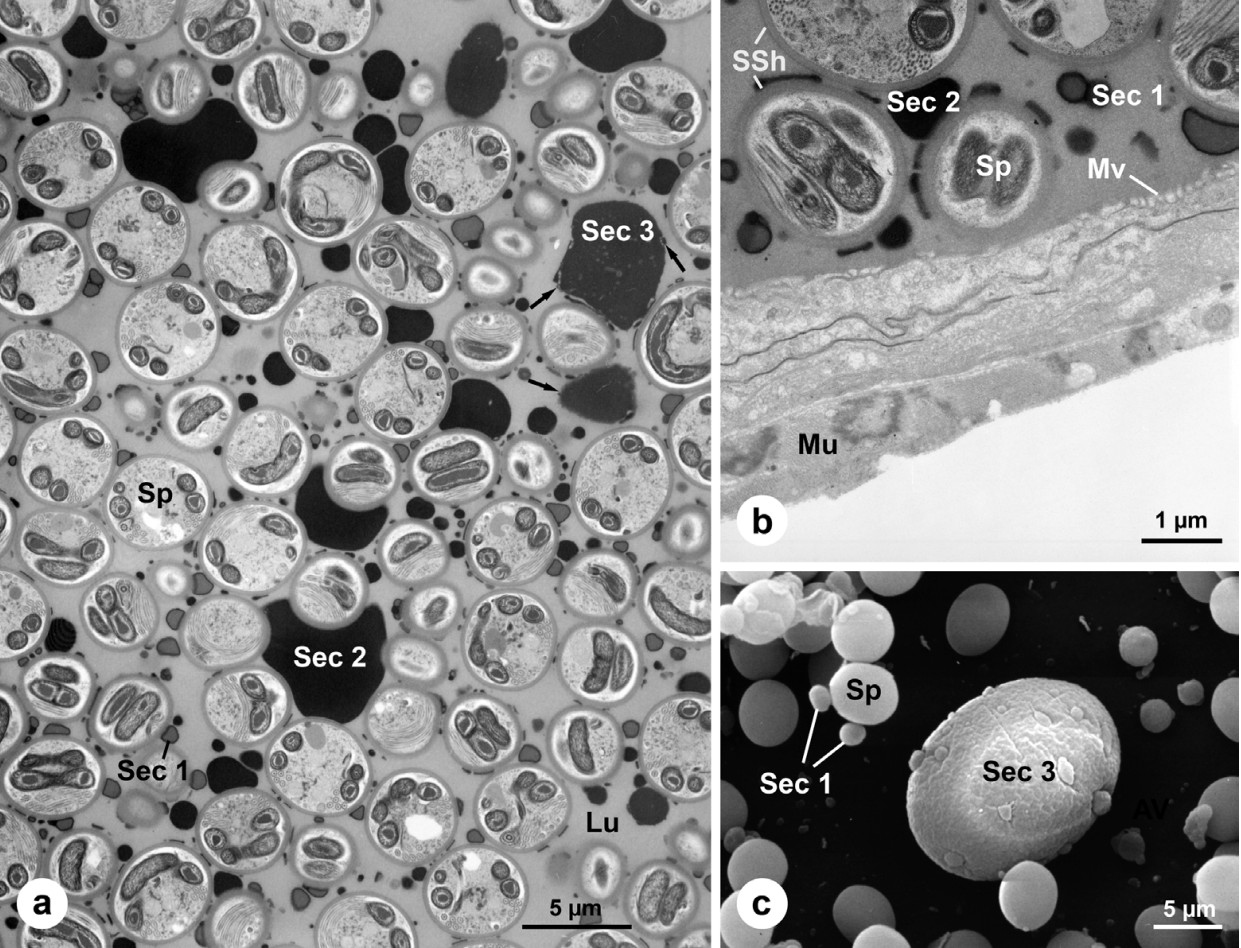
**Ultrastructure of the vas deferens of *Pholcus phalangioides***. (a): The spermatozoa are embedded in a dense secretion matrix. Between the spermatozoa three kinds of secretion droplets are visible. The secretion droplet type 3 is characterized by an irregular surface which sometimes includes other particles (arrows). (b): The epithelium of the vas deferens is very flat and bears microvilli. It is basally bordered by a thin muscle layer. Note the thick secretion sheath which surrounds each spermatozoon. (c): Scanning electron micrograph of the seminal fluid. Note the irregular surface of the secretion droplet type 3 and the differences in size of spermatozoa and secretions. *Lu*, lumen; *Mu*, muscles; *Mv*, microvilli; *Sec1–3*, secretions of droplet type 1–3; *Sp*, spermatozoon; *SSh*, secretion sheath.

In contrast to subadult males, the vasa deferentia possess a narrow lumen filled with spermatozoa and different secretions over their entire length (Figs. 4b,d). Here, the secretion matrix has a bright green color similar to that of the testis (Figs. 4b,d). Furthermore, there are also different red, roundish secretions of varying sizes (Fig. 4d, compare also Fig. 6a).

Close to the genital opening the thin vasa deferentia fuse and form the ductus ejaculatorius, which is characterized by an extensive lumen with large amounts of spermatozoa and secretions (Fig. 4d). The epithelium near the genital opening and towards the ductus ejaculatorius is prismatic and apically bordered by cuticle (Fig. 4e).

Near the ductus ejaculatorius, epiandrous glands are present which open into small spigots anterior to the genital opening (Fig. 4e). These glands are characterized by large lumina and a prismatic epithelium (Fig. 4e). The secretion of the glands has a granular green appearance as seen in the duct of the epiandrous spigots (Fig. 4e). The lumen of the gland appears empty, though this may be an artifact.

### Spermatogenesis and Ultrastructure of the Male Genital System

The spermatozoa of *P. phalangioides *are characterized by several unique features, as pointed out in detail by Alberti and Weinmann [20]. Apart from additional details, we provide information on the relationship between sperm and secretions in the male genital system.

Early stages of spermatogenesis are characterized by a large spherical nucleus surrounded by a manchette of microtubules, an acrosomal vacuole on its anterior pole and an axonemal basis, which migrates into an indentation at the posterior pole of the nucleus. In this early stage, the manchette of microtubules and the nuclear surface possesses a unique arrangement. As seen in cross-sections, the microtubules are not densely packed and the nuclear envelope shows a slightly wave-like outline (Fig. 5a). The quadrangular acrosomal vacuole is tightly connected to the cell membrane, which is indented in this region (Fig. 5a). Within the cytoplasm annulate lamellae are present (Fig. 5a).

During spermiogenesis the chromatin condenses and the nucleus strongly elongates (Fig. 5b). A thin helical band of nuclear material covered by microtubules surrounds the nucleus over its entire length (Figs. 5b,c). The acrosomal vacuole elongates during spermiogenesis, and finally possesses a cylindrical shape (Fig. 5d). A considerable amount of cytoplasm accumulates at the posterior end of the spermatids, forming a big droplet (Figs. 5b,d).

Within the lumen of the testis, only coiled spermatozoa are found embedded in a secretion matrix, which is likely produced by the extensive somatic cells (Figs. 5e,f). The extensions of the somatic cells surround the cysts of spermatids. Apically, the somatic cells bear long microvilli and contain many vesicles, indicative of strong secretory activity (Fig. 5f). Several secretion droplets are visible between the spermatozoa (Figs. 5e,f). The large secretion droplets seem to be produced through fusion of smaller droplets, as indicated by the irregular ring of loose material around the electron-dense center of the droplet (Fig. 5f, arrows).

Within the vas deferens the spermatozoa are densely packed and embedded in a homogenous secretion matrix (Fig. 6a). Each single spermatozoon is surrounded by a secretion sheath forming a so-called cleistosperm (Figs. 6a,b). The secretion sheath is produced within the vas deferens since it is absent in the testis (Fig. 5e). Within the secretion matrix three different kinds of secretions are present (Fig. 6). The first type of secretion consists of small droplets which are densely distributed and characterized by a bright center and a dark border. The second kind is a large electron-dense secretion droplet with a very irregular shape. These droplets often partially surround the spermatozoa (Fig. 6a). The third and rarest type of secretion is a droplet which appears less electron-dense. It is characterized by a heterogeneous content and an irregular surface to which particles may adhere or are partly embedded (Fig. 6c). The epithelium of the vas deferens is flat and bears microvilli in its apical region. A muscle layer surrounds the vas deferens (Fig. 6b).

## Discussion

### General Organization of the Male Genital System

The male genital system of *P. phalangioides *is characterized by thick tube-like testes and thin convoluted vasa deferentia which fuse distally to form the ductus ejaculatorius. This organization is reported from many species of different families, e.g., Cybaeidae, Theridiidae and Agelenidae, and seems to be the general condition in araneomorph spiders (see background). However, theraphosid spiders show no distinct separation between testis and vas deferens [[14]; PM personal observation] and in theraphosid as well as mesothelid spiders the testes themselves are convoluted [[14,22]; PM personal observation]. In some species, e.g., the cybaeid *Argyroneta aquatica*, the testes are curved but distinct from the vasa deferentia [11]. Petrunkevitch [23] suggested that the general organization of the male genital system is not useful for phylogenetic consideration, but the few data available indicate a potential for systematic interpretation. For example, in all theridiid spiders studied thus far, the vasa deferentia lead into a roundish vesicula seminalis (=ductus ejaculatorius?), the storage site of the mature spermatozoa, which is connected via an unpaired duct with the genital opening [[13]; PM personal observation]. Considering the information available to date, it is at least misleading to take the theraphosid type of male genital system as representative for spiders [24,25].

### Development of the Male Genital System

In *P. phalangioides*, spermiogenesis starts several weeks before the last molt and continues in the adults. In the subadult males approximately four weeks before the final molt, the lumina of the testes are very thin and only a small amount of the dense secretion matrix is observable. The secretory activity increases within the last two weeks of the subadult stage. In adults, the lumina are wide and full of secretion and mature spermatozoa. The two different functions of the testis – the production of sperm cells and different kinds of secretion (see below), are evident. Several authors suggested that these secretions are products of degenerated spermatids and the cytoplasmic droplet discarded at the end of spermiogenesis [20,26,27]. However, rough endoplasmic reticulum, Golgi bodies and vesicles in the somatic cells demonstrate that the epithelium itself produces the secretions as was suggested by Alberti et al. [28].

In the subadult stages, the vasa deferentia possess a very extensive lumen in which the mature spermatozoa and secretion from the testes accumulate continuously. The thick epithelium of the vasa deferentia also shows secretory activity. It may be responsible for the formation of the secretion sheath surrounding each spermatozoon, as suggested for other spider species [20,28-32]. In adults, several kinds of secretion are present in the vasa deferentia, whereas in young subadult stages only a dense secretion matrix is present. Thus, it is evident that secretory activity increases in subadults when approaching the final molt. Furthermore, in adult male spiders the epithelium of the vasa deferentia is very thin and secretory activity is low as was stated for the cybaeid *Argyroneta aquatica *[11].

In adult males of *P. phalangioides *cleistospermia and secretions are transferred into the palpal copulatory organ soon after the final molt. Sperm uptake by the male can occur repeatedly after each mating and may require a continuous production of sperm cells. In fact, *P. phalangioides *males mate several times [33] and refill their pedipalp sperm stores after each mating [GU unpublished observation]. To do so successfully, cellar spider males begin sperm production in the subadult phase and continue production throughout adulthood, as demonstrated by our study. In contrast, studies on short lived spider males with only a single mating opportunity showed that males do not refill their sperm stores and probably cease to produce sperm after the final molt [1], instead using their resources for rigorous mate selection [Pauly A, Uhl G, Schneider JM unpublished data].

### Secretory Products

Three different kinds of secretion droplets could be identified in the dense secretion matrices of the testes and vasa deferentia of *P. phalangioides*. According to Romeis [36], the Goldner staining method results in a green coloration of acidic mucosubstances and in red coloration of proteinaceous substances. It cannot be ruled out that the seemingly different kinds of secretion represent different steps of a single secretory pathway. However, since we also found different secretion droplets within the palpal organ, we conclude that different secretory pathways result in a variety of secretions with potentially different functions. Morphologically, the secretions are clearly different from those found in two other spider species, a mesothelid spider [30] and the related pholcid spider *Holocnemus pluchei *[34]. The obvious difference in seminal secretory products in closely related species suggests that there is rapid divergence of seminal fluid substances that may originate from the process of sexual selection. It has been assumed that the rapid evolution of reproductive proteins is a motor in the speciation process [35].

In spiders, sperm and the seminal secretions are not directly transferred into the female genital tract, but taken up into the palpal organ before mating. Within the pedipalps, glandular epithelia adjacent to the sperm storage site seem to be widespread and also occur in *P. phalangioides *and other spider species [[37,38]; Rose W, unpublished data]. It has been assumed that their products serve to release the sperm into the female genital tract during copulation or to serve as a protective matrix for sperm storage within the male palp [e.g., [37,39,40]]. During mating, sperm are transferred to the storage site within the female together with the secretions originating from both genital tract and pedipalp. Finally, inside the female cellar spider, sperm and male secretions encounter secretion that is produced by the female. The only non-morphological investigation existing to date identified proteinaceous substances and glyco- and lipoprotein components in the sperm storage site of virgin female *P. phalangioides *using gel-electrophoretic methods [41].

For spiders, a nutritive and protective function (desiccation and pathogens) has mainly been attributed to either male or female secretions since the sperm cells are often stored for extended periods of time [e.g., [41-45]], but secretions may also serve as lubricants and promote physical uptake or release of sperm inside the male palp (see above). Female secretory products have been suggested to control sperm activation [18,46-48]. However, male secretions may also play an important role in this process.

### Spermatozoa and Spermatogenesis

It has been known since the study of Alberti and Weinmann [20] that *P. phalangioides *possesses very aberrant spermatozoa with several unique characters (e.g., an elongated proximal centriole and a central nuclear canal containing the acrosomal filament), but the most conspicuous character is the band of nuclear material spirally surrounding the nucleus. As shown in the present study, the first signs of this peculiar transformation of the helical band are present in very early spermatid stages: microtubules are loosely arranged around the nucleus resulting in a wave-like outline of the nuclear envelope. The functional implications of the band are still unknown. It may influence the mobility of the spermatozoa or play a role during fertilization. The phylogenetic value of the peculiar spermatozoa of *P. phalangioides *remains uncertain as well, as the sperm ultrastructure of only one further pholcid spider, *H. pluchei*, is known [34,49]. The spermatozoa of these two species however do markedly differ in accord with the present ideas on systematic relationships within the Pholcidae [50]. On the other hand, a helical band of nuclear material is also present in *Spermophora senoculata *[PM personal observation], a species closely related to *P. phalangioides *[50]. It therefore seems that sperm characteristics may be successfully applied successfully in phylogenetic considerations.

## Conclusion

Our study demonstrates that sperm production in males of a long-lived spider species begins weeks before the final molt and continues throughout adulthood. Within the male genital tract, there are at least three different types of proteinaceous secretion droplets apart from a basic matrix secretion containing acidic mucosubstances. The different secretions may serve functions similar to those found in insects, such as oviposition stimulants or manipulators of female receptivity, and clearly warrant future investigation. Moreover, since secretory glands of the female are known to discharge their products into the sperm storage site, their effect on sperm storage, activation and sperm competition should also be investigated. Further, both male and female secretions may interact in as yet unexpected ways. If males have evolved manipulative substances that are against female interests, or if they offer a platform for cryptic female choice, we expect a similar divergence in female secretions and in their interactions with male derived substances.

## Methods

### Light Microscopy (LM)

Males of *P. phalangioides *were collected in houses and cellars within the city of Freiburg i.Br., Germany at an early juvenile stage and reared in the laboratory in individual containers on a diet of fruitflies. During the last intermolt interval, males were classified into two developmental stages according to the external morphology of their developing palpal organs. Young subadult males (stage 1, N = 5) were fixed approximately four weeks before the final molt and older subadult males (stage 2, N = 5) were fixed about two week before the final molt. Adult males (N = 5) were examined one to two days after their final molt.

Male specimens were fixed in Bouin solution according to Gregory [51], dehydrated in a graded series of ethanol or isopropanol and embedded in Paraplast. Sections (7 μm) were made with a Reichert 1150 Autocut microtome, stained according to Goldner [52], and mounted with Eukitt medium. Examination was performed with an Olympus BX 60 and pictures were taken with an Olympus DP 10.

### Transmission Electron Microscopy (TEM)

Elder male specimens (N = 2) were dissected in 0.1 M phosphate buffer supplemented with 1.8% sucrose. The isolated genital system was fixed in 2.5% glutaraldehyde in the same buffer followed by postfixation in buffered 2% osmium tetroxide. After washing, the tissue pieces were dehydrated in graded ethanols and embedded in Spurr's resin [53]. Ultrathin sections (50 nm) were made with a Leica ultramicrotome and stained with uranyl acetate and lead citrate [54]. Examination was performed with a Zeiss EM 10A electron microscope.

### Scanning Electron Microscopy (SEM)

The isolated genital system of elder males (N = 3) was split open in a droplet of phosphate buffer (see above) with thin needles on glass coverslips covered with 1% poly-L-lysin. After 10 min sedimentation the adhering material was fixed in 2.5% glutaraldehyde buffer for 1 h at 4°C. Samples were then rinsed in buffer and post-fixed in buffered 1% osmium tetroxide, dehydrated in ethanol, dried in a Balzers CPD 030 critical point dryer, coated with gold in a Balzers MED 010 sputtering device and examined in a Philips XL20 scanning electron microscope.

## Authors' contributions

PM performed the ultrastructural studies and prepared the manuscript. GU performed the histological studies and helped prepare the manuscript. Both authors read and approved the final manuscript.
